# Bacterial conversion of depolymerized Kraft lignin

**DOI:** 10.1186/s13068-019-1397-8

**Published:** 2019-03-16

**Authors:** Krithika Ravi, Omar Y. Abdelaziz, Matthias Nöbel, Javier García-Hidalgo, Marie F. Gorwa-Grauslund, Christian P. Hulteberg, Gunnar Lidén

**Affiliations:** 1 Department of Chemical Engineering, Lund University, P.O. Box 124, 221 00 Lund, Sweden; 2 Department of Chemistry, Applied Microbiology, Lund University, P.O. Box 124, 221 00 Lund, Sweden; 3 Present Address: Australian Institute for Bioengineering and Nanotechnology (AIBN), The University of Queensland, Brisbane, QLD 4072 Australia

**Keywords:** Base-catalyzed depolymerization, Indulin AT, Microbial conversion, *Pseudomonas*, *Rhodococcus*, Guaiacol, Biorefineries

## Abstract

**Background:**

Lignin is a potential feedstock for microbial conversion into various chemicals. However, the microbial degradation rate of native or technical lignin is low, and chemical depolymerization is needed to obtain reasonable conversion rates. In the current study, nine bacterial strains belonging to the *Pseudomonas* and *Rhodococcus* genera were evaluated for their ability to grow on alkaline-treated softwood lignin as a sole carbon source.

**Results:**

*Pseudomonas fluorescens* DSM 50090 and *Rhodococcus opacus* DSM1069 showed the best growth of the tested species on plates with lignin. Further evaluation of *P. fluorescens* and *R. opacus* was made in liquid cultivations with depolymerized softwood Kraft lignin (DL) at a concentration of 1 g/L. Size-exclusion chromatography (SEC) showed that *R. opacus* consumed most of the available lower-molecular weight compounds (approximately 0.1–0.4 kDa) in the DL, but the weight distribution of larger fractions was almost unaffected. Importantly, the consumed compounds included guaiacol—one of the main monomers in the DL. SEC analysis of *P. fluorescens* culture broth, in contrast, did not show a large conversion of low-molecular weight compounds, and guaiacol remained unconsumed. However, a significant shift in molecular weight distribution towards lower average weights was seen after cultivation with *P. fluorescens*.

**Conclusions:**

*Rhodococcus opacus* and *P. fluorescens* were identified as two potential microbial candidates for the conversion/consumption of base-catalyzed depolymerized lignin, acting on low- and high-molecular weight lignin fragments, respectively. These findings will be of relevance for designing bioconversion of softwood Kraft lignin.

**Electronic supplementary material:**

The online version of this article (10.1186/s13068-019-1397-8) contains supplementary material, which is available to authorized users.

## Introduction

Lignin is one of the most abundant biopolymers on Earth and constitutes 18–32% [1] of woody biomass by weight and 40% by energy [2]. Its natural abundance, high calorific value and the fact that it is one of the few available renewable sources of aromatic chemicals in nature make it a prime feedstock for targeted valorization towards biofuels, renewable polymer composites, and valuable chemical precursors [3, 4]. Despite this, most large-scale industrial facilities that exploit plant polysaccharides (e.g., from wood) have almost exclusively incinerated the co-produced lignin to generate heat and power for biomass conversion and/or product drying, and have not aimed for higher-value products [5]. The emergence of biorefineries, which predominately convert the carbohydrate part of cellulosic biomass into liquid fuels, will generate substantially more lignin than needed for process heat or for power generation to the operation, which will add to the lignin volumes already generated in the pulp and paper industry. Hence, efforts are underway for lignin conversion into higher-value products [6, 7].

Among the three principal polymers in plants (cellulose, hemicellulose and lignin), lignin has the most heterogeneous and complex composition and structure, which makes its efficient utilization a major technological challenge. The lignin biopolymer is built of phenyl propanoid units which are substituted at different positions and connected together by ether and C–C cross-links/bonds [8, 9]. It is noteworthy that the relative abundance of these building blocks is very different between different types of biomass (e.g., softwood, hardwood and monocots) [10]. In addition, there are different techniques for the separation of lignin from lignocellulosic biomass and the resultant lignin vary in terms of bond structures, building block composition, added functional groups, and molecular weight distribution depending on the separation process. Hence, the term “lignin” is per se not very descriptive.

A central process step needed for biological lignin valorization is the breakdown of the lignin macromolecule into smaller compounds that can be converted by microorganisms [1]. This depolymerization process is essential, as most likely only low-molecular weight compounds (mono- or possibly oligomers) can pass the cellular membranes of microbes and enter the cellular metabolism. The distribution of bond strengths in the different C–O and C–C bonds make the depolymerization quite challenging. Ideally, pretreated lignin streams for bacterial conversion should consist of water-soluble monomers. For chemical depolymerization in particular, char formation and repolymerization of produced phenolic radicals to large water-insoluble complexes, thus, need to be avoided. High concentrations of low molecular compounds are also normally desirable—a value of 100 g/L [5] has been mentioned—although tolerance of the microbe to the monomers will likely limit the allowed process concentrations. Until now, obtained monomeric species in lignin streams are still in the range of only a few g/L [11].

The monomers resulting from chemical depolymerization are quite diverse. In a biological conversion process, it is, thus, important to choose organisms that have the ability to funnel a large range of the lignin-derived aromatic compounds to central intermediates (mainly protocatechuate and catechol) via the upper-funneling catabolic pathways [1]. The principal intermediates are then further channeled to valuable products using natural or genetically engineered microorganisms [12–14]. In previous studies, various microorganisms capable of utilizing lignin/model compounds as a sole source of carbon have been identified, including *Pseudomonas putida*, *Rhodococcus opacus*, *Rhodococcus jostii*, *Amycolatopsis* sp., *Sphingobium* sp., and *Cupriavidus necator* [15–18]. Furthermore, successful proof-of-principle studies have been demonstrated for the microbial bioconversion of lignin from alkaline pretreated corn stover and organosolv lignin into various products such as PHA, muconate and lipids [11, 19–25]. Apart from monomers, the depolymerization of lignin also results in a heterogeneous mixture of oligomers and higher-molecular weight compounds; thus, selecting microorganisms able to break down these larger fragments would allow for a process option where complete lignin depolymerization is not required. This, however, requires the use of organisms which secrete extracellular lignolytic enzymes (e.g., laccases and DyP-type peroxidases).

In the present study, we explore the possibilities of utilizing depolymerized softwood Kraft lignin (Indulin AT) as a substrate for bacterial conversion. Softwood Kraft lignin is potentially available at large quantities from pulp mills in Scandinavia [26]. Its inherent complexity, broad molecular weight distribution and high average molecular weight call for depolymerization, if this feedstock is to be used in bioprocessing. Alkaline depolymerized lignin was used to evaluate nine bacterial species with known aromatic-metabolizing capacity for their ability to grow on plates using depolymerized lignin, with the objective of finding organisms capable of both extracellular lignin breakdown and intracellular aromatic metabolism. Further evaluation of selected strains was carried out in liquid shake flask cultivations using alkaline-treated partly depolymerized lignin as a substrate. Additional cultivations were also made with a mixture of selected model compounds that are representative of monomers found in the depolymerized lignin.

## Materials and methods

### Lignin substrate preparation

Depolymerized lignin for plate screening was obtained from previously prepared samples [27] at the temperatures 190, 220 and 240 °C and flow rates of 5 and 10 mL/min. For liquid cultivation experiments, it was prepared as follows: A commercial Kraft lignin, Indulin AT, which is a pine softwood lignin precipitated from the black liquor of linerboard-grade pulp [28], was obtained as a dry brown powder from MeadWestvaco Corporation (Charleston Heights, SC, USA). This lignin fraction has a typical moisture content of 4.5 wt% and ash content of 3.5 wt%. The feed of the chemical depolymerization was comprised of 5 wt% lignin substrate, 5 wt% NaOH, and 90 wt% deionized water. Vacuum filtration for the reactor feed was performed to ensure that no precipitation could take place in the pump and to counteract clogging problems within the pressure valve post reaction.

### Depolymerization apparatus and operation

The base-catalyzed (chemical) depolymerized lignin was prepared using a bench-scale continuous flow reactor (CFR) as previously described in Abdelaziz et al. [27]. In short, the continuous plug flow reactor system consisted of a Gilson 307 HPLC pump (Middleton, WI, USA) and Salamander tubular CFR reactor (Cambridge Reactor Design Ltd., Cottenham, UK) with a typical volume of 10 mL. The reactor was equipped with a preheater (8 mL) that ensures bringing the reactant mixture to the desired reaction temperatures. The product stream exiting the reactor was further condensed with the aid of a Julabo circulating water bath, and a pressure control valve made from nickel–molybdenum–chromium superalloy (Hastelloy C276; UNS N10276) was installed to maintain the pressure throughout reaction. A filter was connected directly after the condensation step and attached to the inlet of the pressure control valve to protect it from any char depositions. The total operating system volume was about 50 mL. The setup was heated up to a working temperature of 220 °C and pressurized to 120–130 bars before starting. The feed was pumped continuously to the plug flow reactor at a volumetric flow rate of 5 mL/min, which corresponds to a residence time in the heating zone of about 2 min. For the reaction operating parameters, the temperature was adjusted using a Eurotherm temperature controller (Eurotherm, Ashburn, Virginia, USA) and the pressure was tuned through a backpressure regulator (Bronkhorst High-Tech B.V., Ruurlo, Netherlands). After exiting the reaction zone and throttling the product mixture/effluent to atmospheric pressure, the liquid depolymerized product (bio-oil) was continuously collected. Samples were taken for analysis and stored at 4 °C until used in liquid cultivations.

### Bacterial strains

Nine bacterial strains were used in this study. *Pseudomonas putida* KT2440 (DSM 6125), *Pseudomonas fluorescens* (DSM 50090), *Rhodococcus erythropolis* (DSM 43066) and *Rhodococcus opacus* (DSM 1069) were purchased from the Deutsche Sammlung von Mikroorganismen und Zellkulturen, Braunschweig, Germany. *Pseudomonas putida* EM42 was generously provided by Esteban Martínez-García at the Centro Nacional de Biotecnología, Madrid, Spain. Four previously reported isolates (B, C, 9.1 and 19), with tentative identifications, were used. Isolate B (*Pseudomonas* sp. strain B; DSM 104484) and isolate C (*Pseudomonas plecoglossicida* strain C; DSM 104486) were isolated from mature compost samples [29] and isolate 9.1 (*Pseudomonas deceptionensis*) and isolate 19 (*R. erythropolis*) were isolated from sediments of the Baltic Sea [30].

### Culture media and carbon source

M9 medium was used for all the solid and liquid culture experiments. The media consist of M9 salts (containing per L: 6 g Na_2_HPO_4_, 3 g KH_2_PO_4_, 1 g NH_4_Cl, 0.5 g NaCl), 2 mM MgSO_4_, 100 µM CaCl_2_ and 10 mL L^−1^ trace element mixture [31, 32]. All the media components were either autoclaved or sterile filtered. The final pH was tuned to 7.

If lignin or depolymerized lignin (DL) was used as a carbon source, the pH was adjusted approximately to 7 using 50% H_2_SO_4_, before adding it to the M9 medium. At pH 7, lignin or DL was stable at no more than 1 g/L. Henceforth, for liquid culture experiments, a concentration of 1 g/L was used to enable biomass measurements.

If lignin model compounds were used as a carbon source, 50 mM stock solutions were prepared and stored (4 °C) for use within 2 weeks. Model compounds, if not soluble in water, were dissolved using a few drops of 5 N NaOH. The final pH of cultivation media containing lignin model compounds was 7. All the chemicals, reagents and materials used were obtained from either VWR (West Chester, PA, USA) or Sigma-Aldrich (St. Louis, USA), unless specified.

### Growth on plates

Plates were prepared using a final concentration of 1.5% agar in M9 medium. The carbon source utilized was 4 g/L glucose, 5 mM vanillin/guaiacol or 1–5 g/L DL. The prepared plates were stored (4 °C) until further use. For screening of several organisms in a single plate, the plates were equally gridded and each grid was inoculated with a single strain. The inoculated plates were sealed (parafilm) and incubated (30 °C).

The lignin and lignin model compound plates were initially inoculated from a freshly grown glucose plate (in M9 medium). Later, when a few colonies were visible, it was re-streaked on to the fresh plates containing the same carbon source. This was performed to maintain the microbial adaptation to a particular carbon source.

### Liquid culture experiments

Liquid culture experiments were conducted in 250-mL shake flasks containing 50-mL culture media. The carbon source added was either 10 g/L glucose (pre-culture), 5 mM guaiacol, 3 mM each of guaiacol, vanillin and 4-HBA (4-hydroxybenzoic acid), 1 g/L DL supplemented with 5 g/L glucose (high-cell density experiments), or 1 g/L lignin or DL (low-cell density experiments).

For the experiments with DL (or un-processed lignin), the flasks were inoculated using a single colony of the corresponding microorganism from 1 g/L DL plates. Shake flasks with model compounds as a carbon source were inoculated with a fixed amount of biomass, using 10 g/L glucose as pre-culture, to achieve an initial OD of around 0.2 or 0.5. All experiments were carried out in duplicates. The flasks were incubated at 27 °C with agitation (180 rpm). Samples were withdrawn at steady intervals to monitor the biomass density (OD—optical density), change in molecular weight of lignin (SEC—size-exclusion chromatography) and consumption of monomers (UHPLC—ultra-high-performance liquid chromatography).

### Biomass measurements

Biomass growth was measured spectrophotometrically by optical density at 620 nm (OD_620_). The color of Kraft lignin is dark and it significantly affects the absorbance measurements; hence, the culture was centrifuged to remove cells and the obtained supernatant was used as a blank prior biomass measurements. The samples, whenever required, were diluted with water/saline to stay in the linear range of optical density (0.03–0.3), in which case, the supernatant was also diluted with the same factor. Subsequently, the cells were removed by centrifugation (3 min at 12,300*g*) and the supernatants were kept at − 20 °C for UHPLC and SEC analyses.

### Calculation of yield and rates

Growth rate, uptake rate and yield were calculated for *R. opacus* on 5 mM guaiacol. Biomass dry weight was measured at the end of cultivation. This was used to convert the optical density into biomass concentration with a response factor of 0.4. Biomass yield (*Y*_SX_), expressed in both g/g and g/mmol, was calculated using the phase plane plot of biomass produced and substrate utilized. The maximum specific growth rate (*µ*) (1/h) was determined from the plot of natural logarithm of biomass in the broth over time. The specific substrate uptake rate (*q*_Substrate_) was calculated by dividing *µ* with *Y*_SX_.

### UHPLC analysis

The frozen samples were thawed, mixed and filtered (0.2-µm pore size) before analysis. A Waters Acquity UPLC system connected with a photodiode array detector (Waters, Milford, MA, USA) was operated for the analysis of phenolic compounds. The column used for separation was Ethylene Bridged Hybrid C18 with a length of 100 mm, 2.1-mm internal diameter and 1.7-µm particle size. Samples were injected at a volume of 2.5 µL and the temperature of the column was kept at 47 °C. The mobile phase was composed of 3% acetonitrile, 95% water, 2% acetic acid (fraction A) and 85% acetonitrile, 13% water, 2% acetic acid (fraction B).

The analysis method was obtained from Schwarz et al. [33] with minor modifications. A flowrate of 0.6 mL/min was used. The LC gradient elution method was as follows: 100% A at time 0, decreased to 90% A in 5 min, held at 90% A for 2 min and decreased to 25% A in 4.5 min. Following the gradient, the column was washed for 5 min with 100% B and was equilibrated for 5 min with 100% A. The chromatographic system was controlled by Acquity UPLC Console and the data were processed using the Empower 3 software (Waters, Milford, MA, USA).

If unknown peaks appeared as a result of metabolic intermediates excretion, MS–MS analysis [30] was performed to identify and confirm the respective compounds. Later, the identified peaks were quantified using standards in UHPLC.

### Size-exclusion chromatography

The molecular weight distributions (MWDs) of different lignin samples were determined with a size-exclusion chromatography (SEC) system, following an established method [34]. The setup adopted for the size measurements was a Waters 600E high-performance liquid chromatography (HPLC) system (Waters, Milford, MA, USA) equipped with a Waters 2414 refractive index detector, a Waters 486 ultraviolet (UV) tunable absorbance detector, and an analytical column packed with 30 cm of Superdex 30 and 30 cm of Superdex 200 (GE Healthcare, Uppsala, Sweden). The column was operating at ambient temperature and eluted with 125 mM NaOH solution (analytical grade) as mobile phase at a flowrate of 1.0 mL/min. Calibration was carried out using polyethylene glycol (PEG) standards ranging from 400 to 35,000 g/mol in the eluent (Merck Schuchardt OHG, Hohenbrunn, Germany). The samples were diluted at concentrations of 0.5 mg/mL in the eluent and the solutions were filtered using a 0.2-µm filter (Schleicher and Schuell, Dassel, Germany) to get rid of any suspended matter. Finally, about 500 µL from the filtered solution was injected into the SEC system for data acquisition. Due to comparison with incorporated PEG standards, both the molecular weight and the molecular number should be interpreted relatively. UHPLC analysis of fractions collected after SEC revealed the presence of aromatic monomers in the 0.4 kDa peak fraction (Additional file 1: Figure S1). The monomers were subsequently identified and quantified using UHPLC.

## Results

### Bacterial screening on agar plates with depolymerized lignin

To identify bacteria able to grow on depolymerized lignin (DL), growth on plates were tested for nine bacteria previously known to metabolize aromatic compounds or isolated on lignin-media [17, 29, 30, 35, 36]. The lignin samples used for screening—obtained from Abdelaziz et al. [27]—had been depolymerized at two different flow rates (5 and 10 mL/min) and three different temperatures (190, 220 and 240 °C) for each flow rate, i.e., in total six different conditions. The initial concentration of DL used with the bacteria was 5 g/L, but this resulted in no visible growth on the plates for the first 3 weeks. Instead, another set of plate screening experiments was made with a reduced DL concentration (2 g/L). Within the first 2 weeks, colonies were formed by *R. opacus* on almost all plates—although only a few at each plate. After one additional week, some colonies were also seen for a few other organisms (Table 1). The growth on these plates was somewhat ambiguous for some of the organisms, but was clearly visible when the organisms were re-streaked onto fresh plates with 1 g/L DL (not shown).

**Table 1:** Growth of bacterial strains on agar plates with 2 g/L depolymerized lignin in M9 medium

Organisms	5 mL/min						10 mL/min						No. of ‘+’ for each organism
	190 °C		220 °C		240 °C		190 °C		220 °C		240 °C		
	1	2	1	2	1	2	1	2	1	2	1	2	
*R. opacus*	+	+	+	+	+	+	+	+	+	+	+	+	12
*R. erythropolis*	+	−	−	−	+	+	−	+	+	−	−	−	6
*P. fluorescens*	−	+	−	+	+	−	+	−	+	+	+	+	8
*P. putida* KT2440	−	−	−	+	−	−	−	−	−	+	−	+	3
*P. putida* EM42	−	−	+	+	−	+	+	−	+	−	−	−	5
Isolate B	−	−	+	+	−	+	+	−	−	+	−	−	5
Isolate C	−	−	−	+	+	+	+	+	−	+	−	−	6
Isolate 9.1	+	−	−	−	−	+	−	+	−	−	−	+	4
Isolate 19	−	−	−	+	−	+	−	+	+	−	−	−	4
No. of species showing growth on depolymerized lignin with different severities	4		7		8		8		8		4		

The lignin samples (originating from [27]) had been depolymerized with two different flow rates (5 and 10 mL/min) and three different severities (190, 220, 240 °C) for each flow rate. The plates were incubated for 3 weeks at 30 °C. The isolates were previously tentatively reported as *Pseudomonas* sp. (B); *P. plecoglossicida* (C); *P. deceptionensis* (9.1); and *Rhodococcus erythropolis* (19). Biological duplicates are indicated as 1 and 2. (+ growth; − no growth)

### Shake flask experiments with depolymerized lignin

*Rhodococcus opacus* DSM 1069 showed growth on the largest number of plates (12), followed by *P. fluorescens* DSM 50090 (8). These two organisms were chosen for experiments with liquid cultures on DL. In addition, *P. putida* EM42 was included due to the well-known robustness of its parental strain KT2440 [29]. Furthermore, the strain EM42 is streamlined for industrial applications by heavy genome editing and hence of interest as a host organism [37]. Based on the previous work on chemical lignin depolymerization [27], a temperature of 220 °C was chosen for preparation of a larger batch of depolymerized lignin for liquid cultures. The yield of lower-molecular weight lignin compounds is likely lower than at 240 °C but the temperature is further from the coking point (250 °C) [27], and preferable for reasons of operational stability. DL, 1 g/L, produced at a flow rate of 5 mL/min and a temperature of 220 °C was supplemented to M9 medium and used in these experiments. Low inoculum flask experiments (initial OD < 0.01) were conducted with both depolymerized and non-depolymerized lignin. A non-inoculated control flask was also included to monitor potential changes in the background OD due to lignin precipitation.

**Fig. 1:**
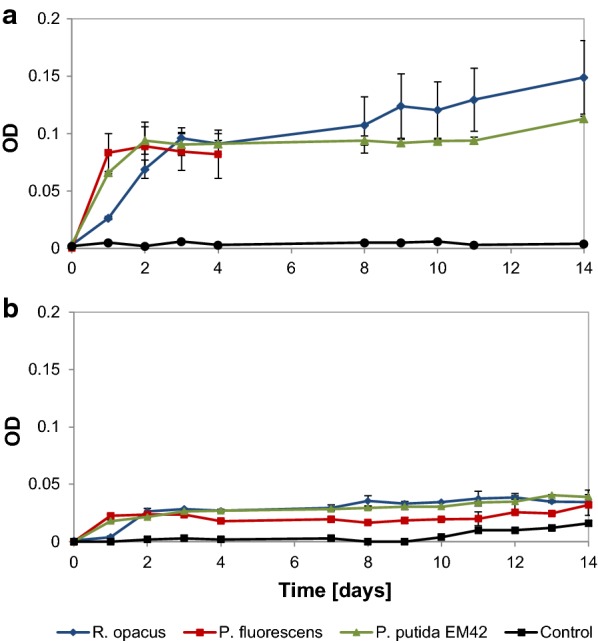
Growth (OD) of *R. opacus*, *P. fluorescens* and *P. putida* EM42 on 1 g/L (**a**) depolymerized (at 220 °C with 2-min residence time) lignin (**b**) non-depolymerized lignin as the only carbon source in M9 medium. A non-inoculated control is shown with a black line. Same scale on the *Y*-axis is maintained for easy comparison between the graphs. All experiments were performed in duplicates

The OD in cultures of *P. fluorescens* reached a plateau after 1 day at value of about 0.1, and similar trend was seen for *P. putida* EM42 and *R. opacus*, but on days 2 and 3, respectively (Fig. 1a). *P. fluorescens* formed aggregates after 4 days and thereafter, it was not possible to measure the cell density by OD; microscopic analysis revealed that the aggregates were bacterial clusters. For all organisms, formation of biofilms was seen at the air–liquid interface of the flask after 4 days. The biofilms were re-suspended before sampling. In the case of the non-depolymerized lignin, less increase in OD was observed, but a small growth was detected for all three organisms in the first 2 days, after which the OD stayed constant until day 14 (Fig. 1b). The maximum OD reached for non-depolymerized lignin was approximately one-third of that obtained with depolymerized lignin. This is in line with the fact that unprocessed lignin has a lower availability of monomers/oligomers for direct consumption than the depolymerized lignin.

**Fig. 2:**
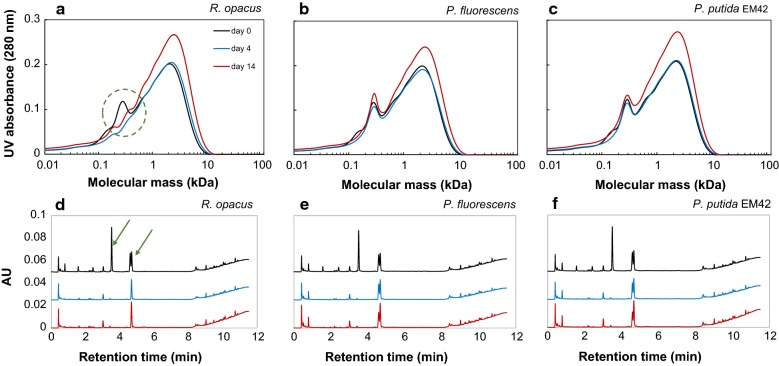
SEC (**a**–**c**) and UHPLC (**d**–**f**) chromatograms of 1 g/L depolymerization lignin on days 0, 4 and 14 after bacterial conversion by *R. opacus*, *P. fluorescens* and *P. putida* EM42. The bacterial cultures, which were inoculated with a single colony, were grown using depolymerized lignin as the only carbon source in M9 medium. In the UHPLC chromatograms (**d**–**f**), the peaks at retention times 1.6, 2.4, 3.5, 4.6 and 4.7 min were identified as 4-HBA, vanillate, vanillin, guaiacol and acetovanillone, respectively. The consumption of low-molecular weight fraction (0.1–0.4 kDa) by *R. opacus* within 4 days is highlighted with a green-dashed circle. The consumption of monomers (vanillin and guaiacol) by *R. opacus* is highlighted with green arrows

From SEC analyses, it was found that the low-molecular weight fraction (0.1–0.4 kDa) of the DL disappeared within 4 days in the cultures with *R. opacus*, indicating a possible consumption of these compounds (Fig. 2a). In the cultures of *P. fluorescens*, the peak for compounds between 0.1 and 0.2 kDa disappeared, and minor effects were possibly seen also for other fractions in the first 4 days (Fig. 2b). In the case of *P. putida* EM42, only compounds between 0.1 and 0.2 kDa disappeared, with no other change in the MWD (Fig. 2c). The peak area and height for the SEC analyses at day 14 were increased for all cultivations, which is probably due to lignin reconfiguration/repolymerization (Fig. 2a–c).

#### Monomer analysis in depolymerized lignin

Ultra-high-performance liquid chromatography analysis showed the presence of several monomers, some of which have previously been identified by supercritical fluid chromatography–mass spectrometry [27, 38]. The previously identified compounds found in the DL were 4-HBA (retention time 1.6 min), vanillate (2.4 min), vanillin (3.5 min), guaiacol (4.6 min) and acetovanillone (4.7 min). These monomer peaks were quantified by UHPLC and the yields based on initial lignin were found to be 3.5, 1.3, 0.95, 0.31, and 0.27 wt%, respectively, for guaiacol, vanillin, acetovanillone, vanillate and 4-HBA, which adds up to the total monomer yield of 6.3 wt%. From the UHPLC chromatograms, it appears that all three organisms consumed 4-HBA, vanillate and vanillin within 4 days (Fig. 2d–f). In addition, *R. opacus* consumed guaiacol as well as an unknown compound that gave a peak eluting at 0.8 min. None of the organisms consumed acetovanillone.

#### Flask experiments with depolymerized lignin and glucose

**Fig. 3:**
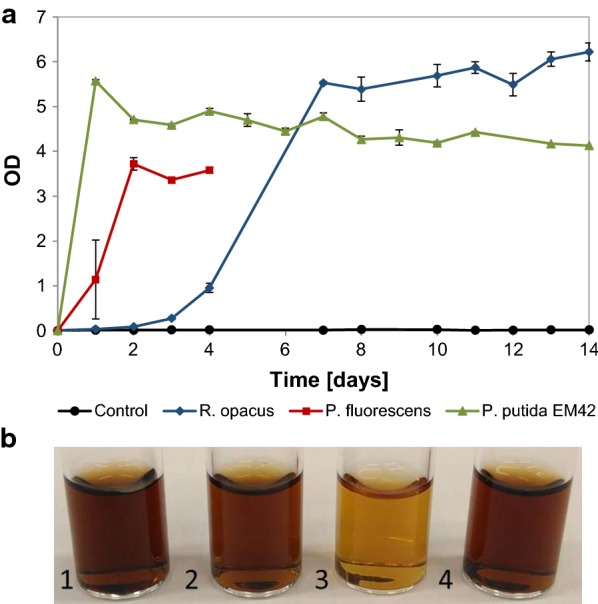
**a** Growth (OD) of *R. opacus*, *P. fluorescens* and *P. putida* EM42 on 1 g/L depolymerized (at 220 °C with 2-min residence time) lignin supplemented with 5 g/L glucose in M9 medium. A non-inoculated control is shown with a black line. **b** Change in media color on day 14 for 1-non-inoculated control; 2-*R. opacus*; 3-*P. fluorescens*; 4-*P. putida* EM42

Experiments were also made in which glucose (5 g/L) was added as a second carbon source, allowing an initial growth phase and a higher biomass concentration for conversion of the DL (1 g/L). All organisms showed growth due to glucose (Fig. 3a), and the fastest growth was found for *P. putida* EM42 which obtained its maximum OD within 24 h. *P. fluorescens* achieved its maximum OD within 2 days. For *R. opacus* the initial growth phase lasted 5–7 days, but the OD kept increasing slowly throughout the entire experiment (Fig. 3a). Similar to the experiments with low cell density, *P. fluorescens* formed aggregates after day 4, and hence it was not possible to monitor the cell density beyond that time. Interestingly, there was a noticeable reduction in media color for *P. fluorescens* (Fig. 3b).

**Fig. 4:**
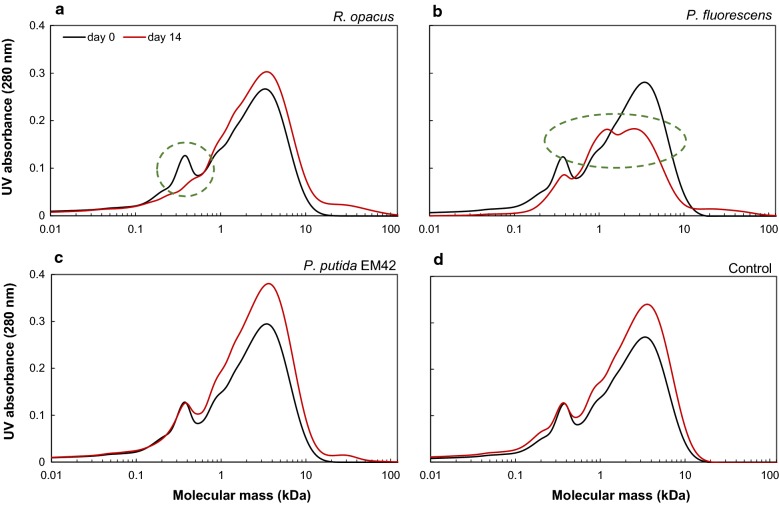
SEC chromatograms of 1 g/L depolymerized lignin on days 0 and 14 after high-cell density bacterial conversion by **a** *R. opacus* **b** *P. fluorescens* **c** *P. putida* EM42 and **d** non-inoculated control in M9 medium. The consumption of low-molecular weight fraction (0.1–0.4 kDa) by *R. opacus* and breakdown of high-molecular weight fraction (1–10 kDa) by *P. fluorescens* are highlighted with green-dashed circles

Size-exclusion chromatography analyses showed that the lower-molecular weight fraction (between 0.1 and 0.4 kDa) was consumed by *R. opacus* (Fig. 4a), similar to the experiments without glucose (Fig. 2a). There was, in contrast, a large change in the SEC chromatograms in the cultivations of *P. fluorescens*. The peak height of the largest fraction (2–7 kDa) was markedly reduced. Part of the lower-molecular weight fraction also remained unconsumed. The SEC chromatogram of *P. putida* EM42 was almost identical to the control, i.e., no major conversion of any fraction was found. An overall increase in peak height between 1 and 10 kDa was observed also for the control, which shows that this change is not due to microbial activity. A slight increase in molecular weight between 15 and 100 kDa was observed for all the inoculated samples (Fig. 4).

**Fig. 5:**
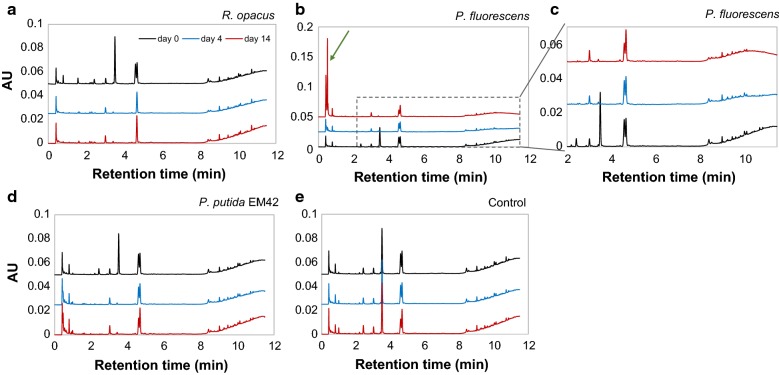
UHPLC chromatograms of 1 g/L depolymerized lignin on days 0, 4 and 14 after high-cell density bacterial conversion by **a** *R. opacus* **b**, **c** *P. fluorescens*, **d** *P. putida* EM42 **e** non-inoculated control in M9 medium. The peaks at retention times 1.6, 2.4, 3.5, 4.6 and 4.7 min were identified as 4-HBA, vanillate, vanillin, guaiacol and acetovanillone, respectively. The occurrence of new large peaks at retention times 0.4–0.5 min for *P. fluorescens* on day 14 are highlighted with a green arrow. For a better visibility of the newly formed peaks on day 14, the chromatograms are arranged in reverse order (bottom–top) for **b**, **c**

The UHPLC chromatograms showed similar patterns for the conversion of the monomers by all the three organisms (Fig. 5) as in the experiments without added glucose (Fig. 2). However, one significant difference was the occurrence of new large peaks at short residence times (0.4–0.5 min) in the experiments with *P. fluorescens* (Fig. 5b).

### Shake flask experiments with aromatic compounds

**Fig. 6:**
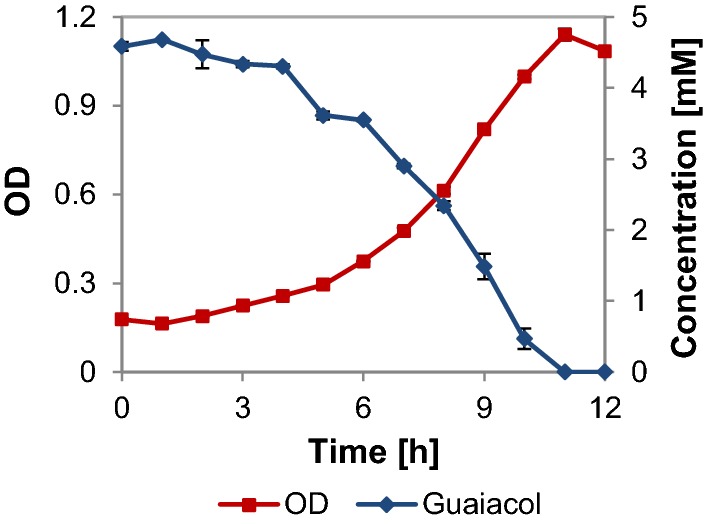
Measured growth (OD) and substrate concentrations for cultures of *R. opacus* grown on guaiacol as the only source of carbon in M9 medium. The standard deviation of duplicate experiments is indicated with error bars

The UHPLC analyses of the shake flask experiments with DL, indicates consumption of guaiacol by *R. opacus* (cf. Figs. 2d and 5a), a rather uncommon trait among microbes. Consumption of guaiacol was, therefore, tested in shake flask experiments of *R. opacus* using guaiacol (5 mM) as the only source of carbon and energy, and growth on guaiacol was indeed confirmed (Fig. 6). Inoculum was taken from agar plates with guaiacol and no lag phase was observed in the shake flask cultures. A specific growth rate of 0.2 (± 0.001) h^−1^ and a biomass yield of 0.7 (± 0.02) g_cdw_/g was obtained and a complete conversion of guaiacol was achieved with a specific conversion rate of 2.4 (mmol/g_cdw_/h).

The main identified low-molecular weight aromatic compounds in the DL, apart from guaiacol, were vanillin, vanillate and 4-HBA (4-hydroxybenzoate). Additional shake flask experiments were made, in which a mixture of 3 mM each of guaiacol, vanillin and 4-HBA served as carbon and energy source for growth of *R. opacus*, *P. fluorescens* and *P. putida* EM42. These three compounds also represent different branches of the upper-funneling/β-ketoadipate pathways [1].

**Fig. 7:**
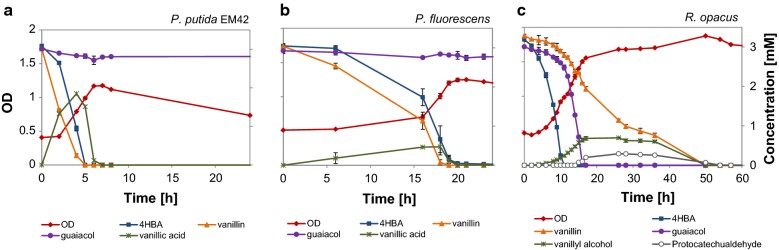
Growth (OD) and consumption of model compounds by **a** *P. putida* EM42, **b** *P. fluorescens*, **c** *R. opacus* in M9 medium. 3 mM each of guaiacol, 4-HBA and vanillin were provided as the only source of carbon. The standard deviation of duplicate experiments is indicated with error bars

The fastest growth was obtained for *P. putida* EM42, which reached its maximum OD in 6 h (Fig. 7a). Vanillin and 4-HBA were converted/consumed almost simultaneously. Vanillin was converted into vanillic acid, which was excreted by the cell, then subsequently taken up (Fig. 7a). Guaiacol, however, remained unconsumed even after 200 h in EM42. *P. fluorescens* showed a somewhat longer lag phase, but after this, vanillin and 4-HBA were simultaneously converted/taken up (Fig. 7b). The conversion of vanillin into vanillic acid was similar to that of *P. putida* EM42 and guaiacol remained unconsumed for 200 h. Multiauxic growth was observed in case of *R. opacus*, which reached its maximum OD in around 50 h (Fig. 7c). The substrate consumption was sequentially starting with 4-HBA, which was consumed in 11 h, and after the depletion of 4-HBA, guaiacol was consumed within 17 h. Interestingly, *R. opacus* converted vanillin into vanillyl alcohol—partly concomitant with the conversion of 4-HBA and guaiacol. A slight excretion of protocatechualdehyde was observed in the medium. The remaining vanillin and the excreted protocatechualdehyde and vanillyl alcohol were all consumed around 50 h (Fig. 7c).

## Discussion

The present study aimed to identify bacterial strains capable of bioconversion of pretreated Indulin AT—a technical softwood Kraft lignin. Kraft lignin is a complex substrate with high molecular weight making it a difficult substrate for direct microbial uptake, and therefore, a previously developed alkaline depolymerization method was employed to break down a part of the higher-molecular weight lignin fragments into compounds of lower molecular weight [27]. The nine bacterial strains/isolates (cf. Table 1) examined here have been previously reported to metabolize lignin-related compounds [17, 21, 29, 35, 36, 39], and fall into two phyla (Proteobacteria and Actinobacteria), with a significant evolutionary distance between them. During the plate screening with DL, an initial concentration of 5 g/L was already inhibitory and hence, no growth was observed within the first 3 weeks. It is well known that several aromatic compounds, e.g., vanillin, are inhibitory to microbial growth and metabolism of many bacteria and yeasts [40]. However, the total concentration of quantified aromatic monomers in the 5 g/L DL was only in the range of few millimoles (2.3 mM), well below the concentrations of the defined medium tested at which *P. putida* EM42, *P. fluorescens*, and *R. opacus* all grew (Fig. 7). This suggests that the inhibition is due to the presence of other unknown toxic compounds present in the depolymerisate. When the concentration was decreased to 2 g/L DL, a few colonies appeared in 2 weeks. An adaption of at least 2 weeks was essential for the organisms to initiate growth on DL plates (Table 1). Upon re-streaking to new lignin plates, the growth was rather quick (3 days).

In liquid cultures of *R. opacus, P. fluorescens* and *P. putida* EM42, the initial increase in OD was due to the uptake of the readily available low-molecular weight lignin compounds in the DL. Later, there was a stationary phase for 8–10 days, after which a slight further growth could be seen (Fig. 1a). Possibly, enzymes for the breakdown of HMW (higher-molecular weight) lignin were expressed during this time and LMW (lower-molecular weight) fragments were obtained. It was not possible to measure the growth of *P. fluorescens* after the first 3 days due to the aggregation of cells in the culture medium. Such behavior of cell aggregation and increased hydrophobicity has previously been observed in *P. putida* CP1 [41, 42] and *P. fluorescens* [21] when grown under stressful conditions. Due to the reduction in size of cells under these conditions, the increase in cell counts has previously been shown to be in disagreement with the cell dry weight and optical density [41].

SEC analyses at day 14 indicated an increase in the average molecular weight (Fig. 2). The main change—at 1–10 kDa—was observed not only for the inoculated cultures, but also for the non-inoculated lignin (cf. Fig. 4d). This phenomenon is, therefore, probably not a result of microbial action, but may be due to instability of lignin fragments, which undergoes reconfiguration and results in higher UV absorbance. The slight increase in molecular weight (in the range of 15–100 kDa) for all the inoculated flasks in high-cell density cultures (Fig. 4a–c) is probably due to the secretion of extracellular laccases. Even though the secreted extracellular enzymes are expected to break down the higher-molecular weight lignin, they are also known for polymerizing phenolic compounds [43]. The balance between the depolymerizing and polymerizing abilities of these enzymes depends on several factors (reaction temperature, origin of enzyme, feedstock structure, etc.) and has been previously reported [44].

The use of cultures with glucose as a co-substrate together with DL revealed the ability of *P. fluorescens* to break down the HMW fraction (Fig. 4b). Both the reduction in color of the DL media (Fig. 3b) and the reduction of peak size in the UPLC chromatograms after 10 min (Fig. 5c) clearly showed that this organism degrades HMW lignin fragments. It was not determined to what extent the degradation products were consumed. Additional peaks appearing in the UHPLC chromatograms indicated that not all the generated compounds were consumed (Fig. 5b). This is in agreement with the reports by Salvachúa et al. on *P. fluorescens* Pf-5, which was found able to depolymerize HMW lignin, but was not able to consume the produced monomers [21]. In contrast to a few previous reports on the ability of *P. putida* KT2440 to breakdown HMW lignin [20, 21], no such behavior was observed for *P. putida* EM42 strain, despite its genetic similarities with KT2440 [37]. This could be due to the different lignin source used in this study, since the lignin obtained from corn stover differs much from the technical lignin (Indulin AT) in terms of the structure, primary building blocks and successive breakdown products [45, 46]. *R. opacus* has previously been reported to break down HMW corn stover lignin to some extent, but a larger conversion was obtained with *R. jostii* in the same study [24]. Co-fermentation using both these strains, furthermore, proved to increase the conversion significantly [24].

Laccases and peroxidases are known to be responsible for higher-molecular-weight-lignin breakdown [47, 48]. Hence, BLAST (Basic Local Alignment Search Tool) searches [49] were performed using well-characterized DyP-type peroxidases [50, 51] and laccases [52] against the genome of *P. fluorescens* (taxid: 294), *P. putida* KT2440 (taxid: 160488) and *R. opacus* (taxid: 37919) to discover, if there are any putative enzymes responsible for lignin breakdown (Additional file 1: Tables S1 and S2). Two enzymes from the subfamily of DyPA and three from DyPB were selected from *R. jostii* RHA1 and *P. fluorescens* Pf-5 [16, 53, 54]. As the enzymes from subfamily DyPC and DyPD were not found/annotated either in *Pseudomonas* or *Rhodococcus* group of organisms, those were selected from the well-studied *Amycolatopsis* sp. 75iv2 and *Bjerkandera adusta,* respectively [50, 55]. Additionally, two laccases were selected, one from *P. fluorescens* and another from *R. opacus* PD630. It seems that laccases, DyPA and DyPB, are widely present (note the absence of DyPA in KT2440) in all the three organisms used in this study (Additional file 1: Tables S1 and S2). However, the inability of *R. opacus* and *P. putida* to depolymerize the HMW lignin to the expected level might be due to the inefficiency of enzyme secretion or due to the lack of gene expression in the given environment. *P. fluorescens* was the only organism in which proteins similar to DyPC and DyPD were found (Additional file 1: Table S1). Steady-state enzyme kinetics of the DyP subclasses has revealed that C- and D-type DyPs have higher peroxidase activities than A- and B-type DyPs [54], possibly explaining the much more efficient degradation of high-molecular weight lignin fractions by *P. fluorescens* than by the other organisms studied.

The growth of organisms on the mixture of model compounds (guaiacol, vanillin and 4-HBA) was in good agreement with their growth on DL—in terms of the consumption of monomers. Depending on the origin of lignin, its mode of separation and the method of depolymerization, very different low molecular compound distributions can be obtained. Previously identified compounds in the depolymerized softwood lignin samples using the depolymerization method employed here have been 4-HBA, vanillate, vanillin and guaiacol [38]. Similar compounds were also reported in other related substrates (Additional file 1: Table S3). These compounds belong to different branches of the upper-funneling pathways, which mostly converge to either protocatechuic acid or catechol that eventually enters the beta-ketoadipate pathway and promotes cell growth [56]. In this study, 4-HBA, vanillin and vanillate were consumed by all the three organisms, shown both from liquid cultures on DL and in model media (M9). 4-HBA, which belongs to the *p*-coumaryl branch of the upper-funneling pathway, has been reported to be converted by a number of microbes, including *Acinetobacter baylyi, Cupriavidus necator, P. aeruginosa*, and is apparently not very toxic [29, 57–59]. In contrast, vanillin and guaiacol (belonging to the coniferyl branch of the funneling pathway) have been reported to be quite toxic to many organisms [60–65]. Microbial detoxification mechanisms of vanillin are related to conversion of its aldehyde group either by oxidation into vanillic acid or by reduction into vanillyl alcohol [66]. The conversion of vanillin towards the less toxic intermediate vanillic acid is common in *Pseudomonas* species [29, 67]. The vanillic acid is—possibly following a period of excretion and accumulation in the medium—further converted into protocatechuic acid entering the beta-ketoadipate pathway. This was observed in our liquid cultivations for *P. fluorescens* and *P. putida* EM42 (Fig. 7a, b). Strong effects on gene expression have been reported when vanillin was provided as a sole carbon source to *P. putida* KT2440. The expression of more than 600 genes was changed, including not only the beta-ketoadipate pathway but also the central energy metabolism and genes associated with solvent tolerance [68].

Interestingly, in our study, *R. opacus* converted vanillin into vanillyl alcohol in the presence of other carbon sources (guaiacol and 4-HBA) (Fig. 7c). The detoxification of vanillin to vanillyl alcohol is mainly reported not only in yeasts [64], but also in some bacterial species such as *Gluconacetobacter xylinus*, which converted vanillin (0.5 mM) into vanillyl alcohol at 80% yield, in the presence of 25 g/L glucose [63]. In the current study, the excretion of vanillyl alcohol stopped after exhaustion of 4-HBA and guaiacol and the remaining vanillin was metabolized within 50 h. Enzymes responsible for vanillin degradation (vanillin dehydrogenase, vanillate *O*-demethylase and vanillyl-alcohol oxidase) have been identified in *Rhodococcus*, but during growth on vanillin, vanillate *O*-demethylase was found to be more upregulated than vanillin dehydrogenase [69]. The inefficient expression of *vdh* (vanillin dehydrogenase) is a possible reason for the detoxification of vanillin to vanillyl alcohol instead of the more common vanillic acid. The slight generation of protocatechualdehyde could be due to the action of the guaiacol demethylase system, which is likely to be highly expressed in this mixture. This system may recognize vanillin with low affinity, remove its methoxy moiety and give rise to small amounts of protocatechualdehyde.

Guaiacol is one of the main depolymerization products in alkaline treatment of softwood lignin [38]. *R. opacus* was the only organism tested which showed growth on guaiacol plates (Additional file 1: Table S4). When grown in liquid cultures, the growth rate on guaiacol was 0.2 h^−1^, which is slightly higher than the growth of the same strain on vanillate (0.127 h^−1^) and 4-HBA (0.126 h^−1^) [17]. Some bacterial species have previously been reported to be able to grow on guaiacol [70, 71] or possess genes related to guaiacol breakdown [72]. Purification and characterization of cytochrome P450 involved in guaiacol demethylation have been reported in *Moraxella* sp., *R. rhodochrous* and *Amycolatopsis* sp. ATCC 3116 [73–75]. A partial N-terminal amino acid sequence of cytochrome P450 responsible for the conversion of guaiacol into catechol was identified for the first time in *R. rhodochrous* [74]. This 21 amino acid-long sequence was used to find the corresponding protein (UniprotKB: W4A0P3) in *R. rhodochrous* (ATCC 21198), which was subsequently blasted against the genome of *R. opacus* (taxid: 37919) and an identity of 81% was found, which strongly indicates the presence of a similar protein. When the same enzyme was blasted against the genome of KT2440 (taxid: 160488), no similar protein was found and with *P. fluorescens* (taxid: 294), only a protein with low identity (28%) was found. *Pseudomonas* sp. strain PN-1 has been reported to convert guaiacol into catechol under *anaerobic* conditions [76]. It was suggested that the anaerobic demethylation system of this species has a broader specificity in the degradation of lignin molecules than the aerobic enzyme [76]. *P. putida* KT2440 possesses genes encoding proteins necessary to metabolize catechol [18], and recently the cloning of genes responsible for the guaiacol-demethylating cytochrome P450 has enabled growth on guaiacol [72, 77]. As the softwood lignin contains a significant amount of G-type lignin, the utilization of guaiacol remains essential.

## Conclusions

Lignins of different origin vary significantly in their composition, and the fragments generated after depolymerization are strongly affected by the type and severity of the treatment. Softwood Kraft lignin is a rather complex substrate, which has been less studied than corn stover lignin for biological valorization. Here, microbial conversion of alkaline-treated Indulin AT—a softwood-based Kraft lignin—was demonstrated. Bacterial growth on the pretreated (partly depolymerized) lignin was clearly better than the growth on unprocessed lignin. Of the bacterial strains tested, *R. opacus* was able to consume most of the LMW compounds and HMW lignin was also converted to some extent. *P. fluorescens* showed a considerable ability for breakdown of HMW lignin. Even more interesting was the ability of *R. opacus* to consume guaiacol, which is the main monomer present after the depolymerization of softwood-based lignin. The yields of monomeric compounds obtained after depolymerization of softwood Kraft lignin typically amount to only a few percent, and this would likely need to be increased to enable commercial processes. However, the organisms of this study are certainly of interest—either as potential host organisms, or sources of genetic material, in development of microbes for the production of fine/bulk chemicals from softwood lignin.

## Additional file

**Additional file 1: Figure S1.** (a) The SEC chromatogram of 1 g/L depolymerized (at 220 °C, 5 mL/min) lignin. Red-dotted lines represent the fractions collected. The collected fraction at 95–108 minutes corresponds to the 0.2–0.4 kDa peak in the SEC chromatograms calibrated with PEG standards. (b) UHPLC chromatograms of the fractions obtained from SEC. The peaks in the fraction 95–108 minutes correspond to aromatic monomers (Vanillin-3.5 min; guaiacol-4.6 min; acetovanillone-4.7 min). **Table S1.** Results of homology BLAST with the previously well-characterized DyP proteins against the genome of the organisms used in this study. *P. putida* EM42 strain used in this study is the modified version of KT2440 and hence, the genome of KT2440 (parental strain) was used for BLAST searches. Proteins with identity more than 75 % are emphasized in green. Proteins that were found absent and the ones with less than 30 % query are highlighted in red. *P. fluorescens* highlighted in blue is the only organism with proteins similar to DyPC and DyPD. **Table S2.** Results of homology BLAST with laccases against the genome of the organisms used in this study. *P. putida* EM42 strain used in this study is the modified version of KT2440 (parental strain) and hence, the genome of KT2440 was used for BLAST searches. Proteins with identity more than 75 % are emphasized in green. **Table S3.** Some studies reporting guaiacol, vanillin and related compounds in depolymerized lignin. **Table S4.** Growth of bacterial strains on agar plates with 5 mM vanillin/guaiacol as the only source of carbon in M9 medium, incubated for 2 weeks at 30 °C. The isolates were previously tentatively reported as *Pseudomonas* sp. (B); *P. plecoglossicida* (C); *P. deceptionensis* (9.1); and *Rhodococcus erythropolis* (19). Duplicate experiments were performed and the growth results were similar (++ abundant growth; + growth; − no growth). *R. opacus* was the only organism to show growth on guaiacol (highlighted).

## Abbreviations

DL: depolymerized lignin
SEC: size-exclusion chromatography
OD: optical density
MWD: molecular weight distribution
UHPLC: ultra-high-performance liquid chromatography
UV: ultraviolet
LMW: low molecular weight
HMW: high molecular weight
BLAST: basic local alignment search tool
CFR: continuous flow reactor
4-HBA: 4-hydroxybenzoate
PEG: polyethylene glycol
HPLC: high-performance liquid chromatography
