# Supplementary material for: Bacterial conversion of depolymerized Kraft lignin
Source: Biotechnol Biofuels. 2019 Mar 16;12:56. doi: 10.1186/s13068-019-1397-8 (PMC6420747; doi:10.1186/s13068-019-1397-8)
Supplement: Supplementary file 1 — Additional file 1: Figure S1. (a) The SEC chromatogram of 1 g/L depolymerized (at 220 °C, 5 mL/min) lignin. Red-dotted lines represent the fractions collected. The collected fraction at 95–108 minutes corresponds to the 0.2–0.4 kDa peak in the SEC chromatograms calibrated with PEG standards. (b) UHPLC chromatograms of the fractions obtained from SEC. The peaks in the fraction 95–108 minutes correspond to aromatic monomers (Vanillin-3.5 min; guaiacol-4.6 min; acetovanillone-4.7 min). Table S1. Results of homology BLAST with the previously well-characterized DyP proteins against the genome of the organisms used in this study. P. putida EM42 strain used in this study is the modified version of KT2440 and hence, the genome of KT2440 (parental strain) was used for BLAST searches. Proteins with identity more than 75 % are emphasized in green. Proteins that were found absent and the ones with less than 30 % query are highlighted in red. P. fluorescens highlighted in blue is the only organism with proteins similar to DyPC and DyPD. Table S2. Results of homology BLAST with laccases against the genome of the organisms used in this study. P. putida EM42 strain used in this study is the modified version of KT2440 (parental strain) and hence, the genome of KT2440 was used for BLAST searches. Proteins with identity more than 75 % are emphasized in green. Table S3. Some studies reporting guaiacol, vanillin and related compounds in depolymerized lignin. Table S4. Growth of bacterial strains on agar plates with 5 mM vanillin/guaiacol as the only source of carbon in M9 medium, incubated for 2 weeks at 30 °C. The isolates were previously tentatively reported as Pseudomonas sp. (B); P. plecoglossicida (C); P. deceptionensis (9.1); and Rhodococcus erythropolis (19). Duplicate experiments were performed and the growth results were similar (++ abundant growth; + growth; − no growth). R. opacus was the only organism to show growth on guaiacol (highlighted). [file 13068_2019_1397_MOESM1_ESM.pdf]

Additional file 1

Bacterial conversion of depolymerized Kraft lignin

Krithika Ravi,[a] Omar Y. Abdelaziz,[a] Matthias Nöbel,[a,c] Javier García-Hidalgo,[b] Marie F. Gorwa-Grauslund,[b] Christian P. Hulteberg[a] and Gunnar Lidén[a,*]

[a]Department of Chemical Engineering, Lund University, P.O. Box 124, SE-221 00 Lund, Sweden

[b]Department of Chemistry, Applied Microbiology, Lund University, P.O. Box 124, SE-221 00 Lund, Sweden

[c] Present address: Australian Institute for Bioengineering and Nanotechnology (AIBN), The University of Queensland, Brisbane, QLD 4072, Australia

*Corresponding author: gunnar.liden@chemeng.lth.se

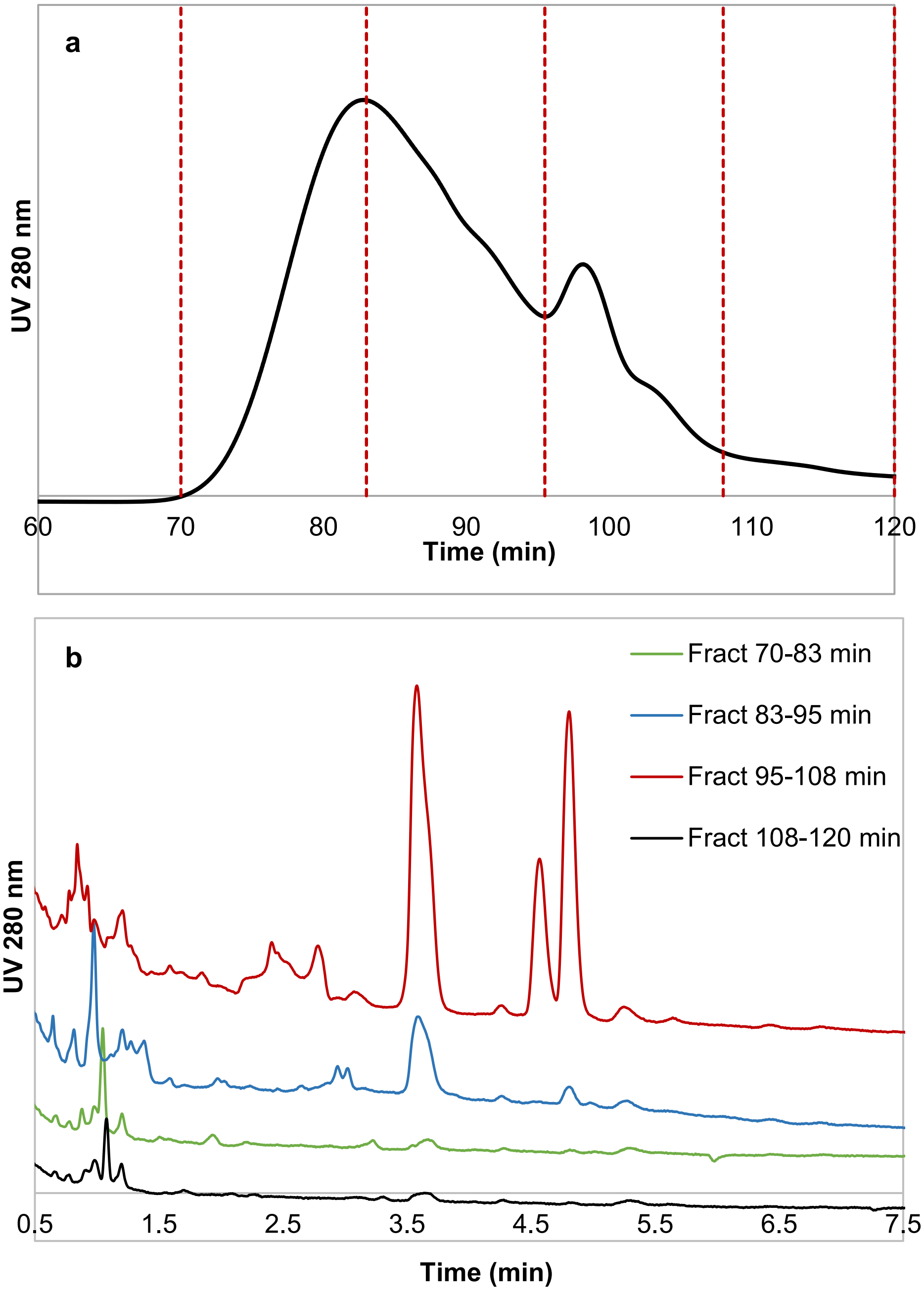

Figure S1: (a) The SEC chromatogram of 1 g/L depolymerized (at 220 °C, 5 mL/min) lignin. Red dotted lines represent the fractions collected. The collected fraction at 95-108 minutes corresponds to the 0.2-0.4 kDa peak in the SEC chromatograms calibrated with PEG standards. (b) UHPLC chromatograms of the fractions obtained from SEC. The peaks in the fraction 95-108 minutes correspond to aromatic monomers (Vanillin-3.5 min; guaiacol-4.6 min; acetovanillone-4.7 min).

Table S1: Results of homology BLAST with the previously well-characterized DyP proteins against the genome of the organisms used in this study. *P. putida* EM42 strain used in this study is the modified version of KT2440 and hence the genome of KT2440 (parental strain) was used for BLAST searches. Proteins with identity more than 75 % are emphasized in green. Proteins that were found absent and the ones with less than 30 % query are highlighted in red. *P. fluorescens* highlighted in blue is the only organism with proteins similar to DyPC and DyPD.

Well characterized DyP proteins	DyP subfamily/ aa length	Blasted against the genome of organisms used in this study	Resultant protein name	Query cover (%)	Identity (%)	Accession number
Dyp-type peroxidase [*R. jostii* RHA1] **UniProtKB: Q0S4I5**	A/428	*P. fluorescens* (taxid: 294)	deferrochelatase/ peroxidase EfeB	97	36	WP_015635791.1
		***P. putida* KT2440**	**Amidohydrolase**	**8**	**35**	**WP_010952751.1**
		***R. opacus* (taxid: 37919)**	**deferrochelatase /peroxidase EfeB**	**100**	**98**	**WP_054246035.1**
Dyp-type peroxidase [*P. fluorescens* Pf-5] **UniProtKB: Q4KBM1**	A/436	***P. fluorescens* (taxid: 294)**	**deferrochelatase /peroxidase EfeB**	**100**	**99**	**WP_015635791.1**
		***P. putida* KT2440**	**--**			
		R. opacus (taxid: 37919)	deferrochelatase/ peroxidase EfeB	84	37	WP_020970278.1
Dyp-type peroxidase [*R. jostii* RHA1] **UniProtKB: Q0SE24**	B/350	*P. fluorescens* (taxid: 294)	Peroxidase	90	55	WP_060755404.1
		P. putida KT2440	Peroxidase	63	32	WP_010954130.1
		***R. opacus* (taxid: 37919)**	**Peroxidase**	**100**	**97**	**WP_065492214.1**
Dyp-type peroxidase [*P. fluorescens* Pf-5] **UniProtKB: Q4KAC6**	1B/295	***P. fluorescens* (taxid: 294)**	**Peroxidase**	**100**	**99**	**WP_015636122.1**
		P. putida KT2440	Peroxidase	97	62	WP_010954130.1
		R. opacus (taxid: 37919)	Peroxidase	79	30	WP_012689337.1
Dyp-type peroxidase [*P. fluorescens* Pf-5] **UniProtKB: Q4KA97**	2B/324	***P. fluorescens* (taxid: 294)**	**Hypothetical protein**	**100**	**99**	**WP_015636148.1**
		P. putida KT2440	Peroxidase	67	32	WP_010954130.1
		R. opacus (taxid: 37919)	Peroxidase	95	59	WP_044480584.1
Multifunctional dye peroxidase [*Amycolatopsis sp. 75iv2*] **UniProtKB: K7N5M8**	C/464	***P. fluorescens* (taxid: 294)**	**Hypothetical protein**	**48**	**30**	**WP_054597259.1**
		***P. putida* KT2440**	**--**			
		***R. opacus* (taxid: 37919)**	**--**			
Dye-decolorizing peroxidase [*Bjerkandera adusta*] **UniProtKB: W8ZBJ5**	D/496	***P. fluorescens* (taxid: 294)**	**Hypothetical protein**	**72**	**25**	**WP_054597259.1**
		***P. putida* KT2440**	**--**			
		***R. opacus* (taxid: 37919)**	**--**			

Table S2: Results of homology BLAST with laccases against the genome of the organisms used in this study. *P. putida* EM42 strain used in this study is the modified version of KT2440 (parental strain) and hence the genome of KT2440 was used for BLAST searches. Proteins with identity more than 75 % are emphasized in green.

**Laccases**	**aa length**	**Blasted against the genome of organisms used in this study**	**Resultant protein name**	**Query cover (%)**	**Identity (%)**	**Accession number**
Laccase-2 [*R. opacus* PD630] **UniProtKB: W8HL51**	494	*P. fluorescens* (taxid: 294)	copper resistance system multicopper oxidase	84	34	WP_003193107.1
		P. putida KT2440	copper resistance system multicopper oxidase	87	34	WP_010953188.1
		***R. opacus* (taxid: 37919)**	**multicopper oxidase family protein**	**100**	**100**	**WP_005251383.1**
Multi-copper polyphenol oxidase [*P. fluorescens*] **UniProtKB: A0A293QHC5**	239	***P. fluorescens* (taxid: 294)**	**peptidoglycan editing factor PgeF**	**100**	**100**	**WP_057713252.1**
		***P. putida* KT2440**	**multi-copper polyphenol oxidoreductase**	**98**	**76**	**WP_010951886.1**
		R. opacus (taxid: 37919)	peptidoglycan editing factor PgeF	94	37	WP_020969856.1

Table S3: Some studies reporting guaiacol, vanillin and related compounds in depolymerized lignin

Substrate	Depolymerization method	Major monomers	Reference
Steam treated softwood and hemp lignin	Base-catalyzed depolymerization	Guaiacol, vanillin, catechol	[1]
Organosolv lignin	Base-catalyzed depolymeriztaion	Guaiacol, Syringol, syringaldehyde,	[2]
Spruce lignin	Hydrogenolysis/Extraction	Ethylguaiacol, propylguaiacol,	[3]
Dealkaline lignin	Solid acid catalysts	Vanillin, guaiacyl acetone, homovanillic acid	[4]
Birch wood lignin	Hydrogenolysis with Ni-based catalysts	Propyl guaiacol and propyl syringol	[5]
Organosolv switchgrass lignin	Depolymerization and hydrodeoxygenation with formic acid and Pt/C	Guaiacol, 4-methylguaiacol, homovanillyl alcohol	[6]
Organosolv hardwood lignin	Depolymerization in supercritical carbon dioxide/acetone/water fluid	Guaiacol, 2-methoxy-4-methyl-phenol, syringol	[7]
Kraft lignin	oxidation in aqueous methanol at acidic pH	Vanillin, methyl vanillate	[8]
Kraft lignin (Indulin AT)	Base (NaOH) catalyzed depolymerization at 220 °C	Guaiacol, vanillin, acetovanillone, vanillate, 4-hydroxybenzoate	This study

Table S4: Growth of bacterial strains on agar plates with 5 mM vanillin/guaiacol as the only source of carbon in M9 medium, incubated for 2 weeks at 30 °C. The isolates were previously tentatively reported as *Pseudomonas* sp. (B); *P. plecoglossicida* (C); *P. deceptionensis* (9.1); and *Rhodococcus erythropolis* (19). Duplicate experiments were performed and the growth results were similar (++ abundant growth; + growth; - no growth). *R. opacus* was the only organism to show growth on guaiacol (highlighted).

Organisms	5 mM vanillin	5 mM guaiacol
R. opacus	**+**	**+**
R. erythropolis	-	-
P. fluorescens	++	-
P. putida KT2440	++	-
P. putida EM42	++	-
Isolate B	++	-
Isolate C	-	-
Isolate 9.1	+	-
Isolate 19	-	-

References

1. Lavoie JM, Bare W, Bilodeau M. Depolymerization of steam-treated lignin for the production of green chemicals. Bioresour Technol. 2011;102:4917-4920.
2. Roberts VM, Stein V, Reiner T, Lemonidou A, Li X, Lercher JA. Towards quantitative catalytic lignin depolymerization. Chemistry. 2011;17:5939-5948.
3. Shuai L, Amiri MT, Questell-Santiago YM, Héroguel F, Li Y, Kim H, Meilan R, Chapple C, Ralph J, Luterbacher JS. Formaldehyde stabilization facilitates lignin monomer production during biomass depolymerization. Science. 2016;354:329-333.
4. Deepa AK, Dhepe PL. Lignin Depolymerization into Aromatic Monomers over Solid Acid Catalysts. ACS Catalysis. 2015;5:365-379.
5. Song Q, Wang F, Cai JY, Wang YH, Zhang JJ, Yu WQ, Xu J. Lignin depolymerization (LDP) in alcohol over nickel-based catalysts via a fragmentation-hydrogenolysis process. Energy & Environmental Science. 2013;6:994-1007.
6. Xu W, Miller SJ, Agrawal PK, Jones CW. Depolymerization and hydrodeoxygenation of switchgrass lignin with formic acid. ChemSusChem. 2012;5:667-675.
7. Gosselink RJ, Teunissen W, van Dam JE, de Jong E, Gellerstedt G, Scott EL, Sanders JP. Lignin depolymerisation in supercritical carbon dioxide/acetone/water fluid for the production of aromatic chemicals. Bioresour Technol. 2012;106:173-177.
8. Voitl T, von Rohr PR. Reply to "Comments on 'Demonstration of a Process for the Conversion of Kraft Lignin into Vanillin and Methyl Vanillate by Acidic Oxidation in Aqueous Methanol'". Industrial & Engineering Chemistry Research. 2010;49:3501-3503.
